# Lymph node ratio is a prognostic indicator for locally advanced esophageal squamous cell carcinoma after neoadjuvant immunochemotherapy

**DOI:** 10.17305/bb.2023.9435

**Published:** 2024-02-01

**Authors:** Pengcheng Chen, Liang Wang, Xun Yang, Jifeng Feng

**Affiliations:** 1 Department of Thoracic Surgery, Zhejiang Provincial People’s Hospital (Affiliated People’s Hospital, Hangzhou Medical College), Hangzhou, China; 2 Department of Thoracic Surgery, Zhejiang Cancer Hospital, Hangzhou Institute of Medicine, Chinese Academy of Sciences, Hangzhou, China; 3 The Second Clinical Medical College, Zhejiang Chinese Medical University, Hangzhou, China

**Keywords:** Neoadjuvant immunochemotherapy (NICT), esophageal squamous cell carcinoma (ESCC), pathologic complete response (PCR), overall survival (OS), disease-free survival (DFS), lymph node ratio (LNR)

## Abstract

The lymph node ratio (LNR) is regarded as a prognostic indicator in esophageal cancer (EC), but its applicability to neoadjuvant immunochemotherapy (NICT) in esophageal squamous cell carcinoma (ESCC) remains unexplored. This retrospective study, conducted between 2019 and 2021, analyzed ESCC patients who underwent radical esophagectomy following NICT. Patients were divided into two groups based on their LNR values according to the X-tile software: Low-LNR group (LNR 0%–10%) and High-LNR group (LNR 10%–100%). The association between LNR and clinical outcomes in ESCC after NICT were analyzed. A total of 212 ESCC patients who underwent surgery after NICT were included in this study, among which, 169 (79.7%) and 43 (20.3%) cases were allocated to the Low- and High-LNR groups, respectively. Pathologic complete response (PCR) was observed in 28.3% (60/212) of the overall cohort. Patients in the Low-LNR group demonstrated a significantly improved 3-year overall survival (OS) (81.7% vs 55.3%; *P* < 0.001) and disease-free survival (DFS) (79.9% vs 37.4%; *P* < 0.001). These findings were consistent among those with non-PCR (3-year DFS was 73.7% vs 37.4%; *P* < 0.001, and the 3-year OS was 78.9% vs 55.3%; *P* < 0.001, respectively). High LNR was associated with a 4.013-fold increased risk of relapse and a 7.026-fold elevated risk of death. Compared to the post-neoadjuvant therapy pathologic lymph nodes staging (ypN), LNR exhibited similar prognostic capabilities for DFS and OS. To the best of our knowledge, this study is the first to investigate the prognostic value of LNR in ESCC after NICT, suggesting that LNR may serve as a viable alternative to the ypN stage for prognostication in ESCC patients treated with NICT.

## Introduction

Esophageal cancer (EC) continues to be one of the leading cancer types in the world with a high mortality and morbidity (ranking sixth and seventh in terms of cancer mortality and morbidity, respectively) [1]. Currently, surgical therapy, involving radical resection of the esophagus with lymph node (LN) dissection, is the primary treatment method for EC [2]. However, many patients are diagnosed late and miss the opportunity for surgery [2]. Recently, with the development in multidisciplinary collaboration and the introduction of neoadjuvant therapies (NATs), such as neoadjuvant chemotherapy (NCT) and neoadjuvant chemoradiotherapy (NCRT), the treatment approach for EC has changed offering new possibilities [3, 4]. However, since the real-world study results show a high relapse rate, the outcomes after NAT for EC are still unsatisfactory [5]. Therefore, the exploration for more effective and safe NATs in EC is necessary and urgent.

Immunotherapy, represented by immune checkpoint inhibitors (ICIs), has achieved remarkable results in several studies in recent years, significantly affecting the therapeutic strategies for those with advanced EC [6, 7]. Due to the enormous benefits of immunotherapy in the treatment of the advanced cancer, an emerging hotspot—neoadjuvant immunochemotherapy (NICT)—has garnered increased attention [8]. To date, a variety of real-world evidences demonstrate that NICT is both safe and effective in locally advanced EC [9–11]. As an emerging treatment, however, the prognosis after NICT in EC is still unclear and needs to be further clarified.

Currently, the American Joint Committee on Cancer (AJCC) 8th version of the post-NAT pathologic TNM (ypTNM) staging system is the most widely used tool to access the prognosis of EC after NAT [12]. According to this EC staging system, the post-neoadjuvant therapy pathologic lymph nodes (ypN) stage is determined by the number of positive lymph nodes (PLNs), regardless of the total number of lymph nodes (TLNs) examined. Therefore, the accuracy of the ypN stage might be seriously compromised due to the insufficient number of TLNs [13]. Moreover, several published studies have also shown a positive association between a higher number of TLNs and better prognosis [14, 15]. Therefore, evaluating both the number of TLNs and PLNs is important in cancer treatment.

Recently, the lymph node ratio (LNR), which is the ratio of PLNs to TLNs, has been shown to be an important predictor in a variety of cancers, including EC [16–18]. Additionally, the prognostic role of LNR is also recognized in EC patients who have received NAT [19, 20]. However, its applicability to NICT in esophageal squamous cell carcinoma (ESCC) remains unknown. Therefore, the present study aims to investigate the prognostic value of LNR in relation to NICT in ESCC.

## Materials and methods

### Study design

The medical records of all patients who underwent radical resection after NICT in Zhejiang Cancer Hospital from 2019 to 2021 were collected. The study included ESCC patients aged 18–75 years, who were in clinical TNM stage II-IVA and had undergone radical resection after NICT. Moreover, these patients had not received any other anticancer therapy before NICT. Additionally, patients with any concurrent or previous cancers, hematologic or autoimmune diseases were excluded from this study.

### Treatments and follow-up

NICT was administered every 21 days, and a total of two cycles were performed before surgery. During each cycle, patients received an intravenous administration of either sintilimab (200 mg), camrelizumab (200 mg), tislelizumab (200 mg), nivolumab (3 mg/kg), or pembrolizumab (2 mg/kg) on day 1. Additionally, albumin paclitaxel was given at a dose of 100 mg/m^2^ based to the body surface area on days 1 and 8. Moreover, carboplatin calculated at 5 mg/mL/min based on the area under the curve (AUC), was administered on day 1 [9–11]. In general, the clinical effect evaluation was performed after two cycles of NICT. For patients showing tumor shrinkage, a multidisciplinary discussion was conducted to determine whether to proceed with the NICT or to opt for surgery. Typically, 4–6 weeks after the final NICT cycle, surgery following the Ivor Lewis or McKeown procedure was scheduled. Procedures, including the minimally invasive esophagectomy (MIE) or open esophagectomy (OE) in two- or three-field LN dissection, were carried out [21, 22]. The Clavien–Dindo classification was used to refer to major complications [23]. Staging was based on the 8th AJCC/Union for International Cancer Control (UICC) TNM classification system [12]. The pathological complete response (PCR) was defined as the absence of viable tumor cells in both the resected specimen and all TLNs [24]. To date, no consensus has been reached regarding adjuvant treatment following NICT. According to the CheckMate 577 study, patients could benefit from adjuvant immunotherapy after NCRT [25]. Generally, in our institute, two cycles of adjuvant immunotherapy combined with chemotherapy are administered post-surgery, though this is not mandatory. In addition, for patients exhibiting the postoperative pathology of ypT3-T4a and/or ypN+, adjuvant chemoradiotherapy was also recommended [26, 27]. The final follow-up period was set to be December 2022.

### Lymph node ratio status

The ypN stage analyzed in this study was based on 8th AJCC/UICC staging system [12]. The LNR was calculated as the ratio of the number of PLNs to the TLNs. Using PCR as the dependent variable, the LNR was then categorized into two groups based on the optimal cut-off value of 10%, as determined by the X-tile software. By creating a two-dimensional projection of each potential subpopulation, a graphical method demonstrates the robustness of the association between a biomarker and the outcome. Therefore, for the purpose of further analysis in this study, the High-LNR group (LNR ranging from 10% to 100%) and the Low-LNR group (LNR ranging from 0% to 10%) were investigated.

### Ethical statement

The study was conducted in accordance with the Declaration of Helsinki and approved by the Ethics Committee of Zhejiang Cancer Hospital (IRB-2020-320). Informed consent was signed by each patient in the current study.

### Statistical analysis

Statistical analyses were carried out using the SPSS 20.0, MedCalc 17.6 and R 4.1.2. Continuous variables were analyzed using the Mann–Whitney *U* test or Student’s *t*-test, while categorical variables were analyzed with the chi-square or Fisher’s exact tests. AUCs were performed to better understand the prognostic ability of LNR based on receiver operating characteristic (ROC) curves. Predictors affecting prognosis, including disease-free survival (DFS) and overall survival (OS), were identified by the Cox regression analyses. The survival differences were evaluated using the Kaplan–Meier curves and compared with log-rank tests. All statistical tests were two-sided, and a *P* value < 0.05 was considered to be statistically significant.

## Results

### Patients characteristics

**Figure 1.**
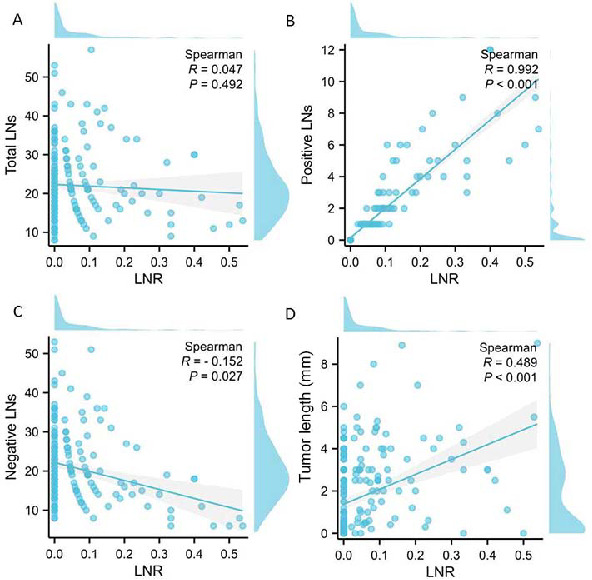
**The correlations between the LNR and different variables.** (A) The correlation between the LNR and the total LNs; (B) The correlation between the LNR and the positive LNs; (C) The correlation between the LNR and the negative LNs; (D) The correlation between the LNR and the tumor length. LNR: Lymph node ratio; LNs: Lymph nodes.

**Figure 2.**
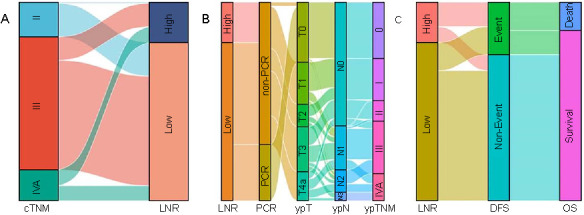
**Sankey diagram analyzing the associations between the LNR and TNM stages.** The diagram is analyzing the association between the LNR and cTNM (A), ypTNM (B), and the prognosis (C). LNR: Lymph node ratio; cTNM: Clinical TNM staging system; ypTNM: Post-neoadjuvant therapy pathologic TNM staging system; PCR: Pathological complete response; ypT: Post-neoadjuvant therapy pathologic primary tumor staging; ypN: Post-neoadjuvant therapy pathologic lymph nodes staging; DFS: Disease-free survival; OS: Overall survival.

A total of 212 ESCC patients, who underwent radical resection after NICT, were included in the current study. The mean age was 63.2 ± 6.7 years, ranging from 47 to 75 years. Among the patients, 20 (9.4%) were female and 192 (90.6%) were male. A total of 181 (85.4%) patients underwent MIE, with the remaining 31 (14.6%) cases undergoing an OE. The mean number of total, positive, and negative LNs were 22.1 ± 8.9 (range: 8–57), 1.14 ± 2.17 (range: 0–12), and 21.0 ± 8.7 (range: 6–53), respectively. The mean LNR was 0.054 ± 0.105 (range: 0–0.538). The correlations between the LNR and total, positive, and negative LNs are shown on Figure 1A–1C. LNR exhibited a positive correlation with the positive LNs (*r* ═ 0.992; *P* < 0.001), and a negative correlation with the negative LNs (*r* ═ −0.152; *P* ═ 0.027). In addition, a positive correlation was found between LNR and tumor length (*r* ═ 0.489; *P* < 0.001; Figure 1D). Among the patients, 12 (5.7%) received immunotherapy with nivolumab, 29 (13.7%) with pembrolizumab, 116 (54.7%) with camrelizumab, 39 (18.4%) with tislelizumab, and 16 (7.5%) with sintilimab. The associations between the LNR and TNM stages were analyzed using a Sankey diagram (Figure 2). The mean follow-up duration was 17 months, ranging from 7 to 36 months. A total of 56 (26.4%) patients experienced relapse, and 30 (14.2%) patients died.

**Table 1:** Clinical and intraoperative characteristics, as well as postoperative complications in ESCC patients grouped by their LNR values, after the NICT

	**Total (*n* ═ 212)**	**Low-LNR (*n* ═ 169)**	**High-LNR (*n* ═ 43)**	***P* value**
*Clinical characteristics*				
Sex (female/male, *n*)	20/192	17/152	3/40	0.745
Age (years, mean ± SD)	63.2 ± 6.7	63.8 ± 6.5	60.8 ± 7.0	0.008
ECOG score (0/1, *n*)	183/29	149/20	34/9	0.121
Smoking history (yes/no, *n*)	146/66	114/55	32/11	0.379
Drinking history (yes/no, *n*)	149/63	115/54	34/9	0.158
BMI (kg/m^2^, mean ± SD)	21.8 ± 2.0	21.7 ± 1.9	22.0 ± 2.6	0.533
Tumor location (*n*):				0.011
- Upper	20	15	5	
- Middle	126	109	17	
- Lower	66	45	21	
Differentiation (*n*):				0.023
- Well	36	33	3	
- Moderate	93	77	16	
- Poor	83	59	24	
Vessel invasion (yes/no, *n*)	23/189	9/160	14/29	<0.001
Perineural invasion (yes/no, *n*)	35/177	21/148	14/29	0.001
Tumor length (cm, mean ± SD)	0.21 ± 0.41	0.15 ± 0.36	0.44 ± 0.50	0.001
Surgical procedure (MIE/OE, *n*)	181/31	146/23	35/8	0.408
PCR (yes/no, *n*)	60/152	60/109	0/43	<0.001
ypT stage (*n*):				<0.001
- T0	64	62	2	
- T1	45	41	4	
- T2	24	18	6	
- T3	48	29	19	
- T4a	31	19	12	
ypN stage (*n*):				<0.001
- N0	132	132	0	
- N1	47	35	12	
- N2	24	2	22	
- N3	9	0	9	
ypTNM stage (n):				<0.001
0	60	60	0	
- I	45	45	0	
- II	22	21	1	
- III	56	32	24	
- IVA	29	11	18	
*Intraoperative characteristics*				
Total LNs (*n*, mean ± SD)	22.1 ± 8.9	21.9 ± 8.4	22.8 ± 10.6	0.542
Positive LNs (*n*, mean ± SD)	1.14 ± 2.17	0.27 ± 0.58	4.56 ± 2.69	<0.001
Negative LNs (*n*, mean ± SD)	21.0 ± 8.7	21.6 ± 8.3	18.3 ± 9.9	0.024
Operating time (min, mean ± SD)	221.9 ± 32.5	218.2 ± 31.0	236.5 ± 34.6	0.001
Blood loss (mL, mean ± SD)	132.9 ± 46.2	127.7 ± 44.0	153.3 ± 49.3	0.001
Stay after surgery (day, mean ± SD)	14.3 ± 6.9	14.0 ± 6.4	15.6 ± 8.6	0.172
*Postoperative complications*				
Respiratory complications (yes/no, *n*)	48/164	36/133	12/31	0.355
Anastomotic leakage (yes/no, *n*)	24/188	18/151	6/37	0.733
Vocal cord paralysis (yes/no, *n*)	23/189	13/156	10/33	0.008
Chylothorax (yes/no, *n*)	5/207	4/165	1/42	0.987

The patients were categorized into two groups based on their LNR values using the optimal cut-off value of 10%, as determined by the X-tile software. These groups were defined as the High-LNR group (with LNR ranging from 10% to 100%) and the Low-LNR group (with LNR ranging from 0% to 10%). LNR: Lymph node ratio; ESCC: Esophageal squamous cell carcinoma; NICT: Neoadjuvant immunochemotherapy; ECOG: Eastern Cooperative Oncology Group; BMI: Body mass index; PCR: Pathological complete response; MIE: Minimally invasive esophagectomy; OE: Open esophagectomy; ypT: Post-neoadjuvant therapy pathologic primary tumor staging; ypN: Post-neoadjuvant therapy pathologic lymph nodes staging; ypTNM: Post-neoadjuvant therapy pathologic TNM staging system; LNs: Lymph nodes.

**Figure 3.**
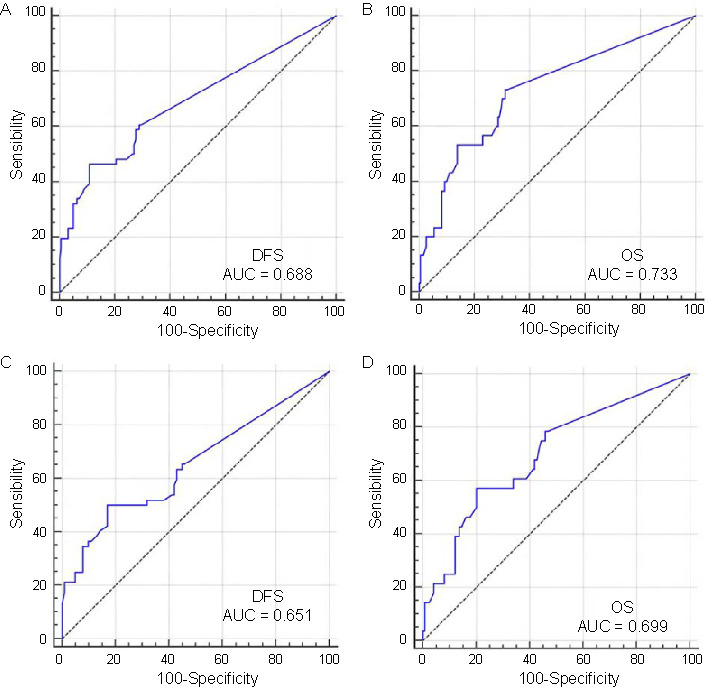
**The predictive value of LNR for DFS and OS.** (A) The predictive value of LNR for the DFS in all cohorts; (B) The predictive value of LNR for the OS in all cohorts; (C) The predictive value of LNR for the DFS in non-PCR cohorts; (D) The predictive value of LNR for the OS in non-PCR cohorts. LNR: Lymph node ratio; DFS: Disease-free survival; OS: Overall survival; PCR: Pathological complete response; AUC: Area under the curve.

### Characteristics of patients grouped based on their lymph node ratio value

The clinical and intraoperative characteristics, as well as the postoperative complications, are shown in Table 1. Regarding the clinical characteristics, the age in the Low-LNR group was higher than that in the High-LNR group (63.8 ± 6.5 vs 60.8 ± 7.0 years; *P* ═ 0.008), while the tumor length was lower in the Low-LNR group compared to the High-LNR group (0.15 ± 0.36 vs 0.44 ± 0.55 cm; *P* ═ 0.001). Moreover, LNR exhibited significant associations with the differentiation, location, neurovascular invasion, and staging. In addition, all patients (60 cases) who achieved a PCR were found within the Low-LNR cohort (*P* < 0.001). In terms of intraoperative characteristics, LNR demonstrated significant correlations to positive (*P* < 0.001) and negative (*P* ═ 0.024) LNs, operating time (218.2 ± 31.0 vs 236.5 ± 34.6 min; *P* ═ 0.001) and blood loss (127.7 ± 44.0 vs 153.3 ± 49.3 mL; *P* ═ 0.001). However, only vocal cord paralysis (*P* ═ 0.008) exhibited a significant difference between the two groups in relation to all complications.

### Predictive value of the lymph node ratio for disease-free survival and overall survival in esophageal squamous cell carcinoma

**Figure 4.**
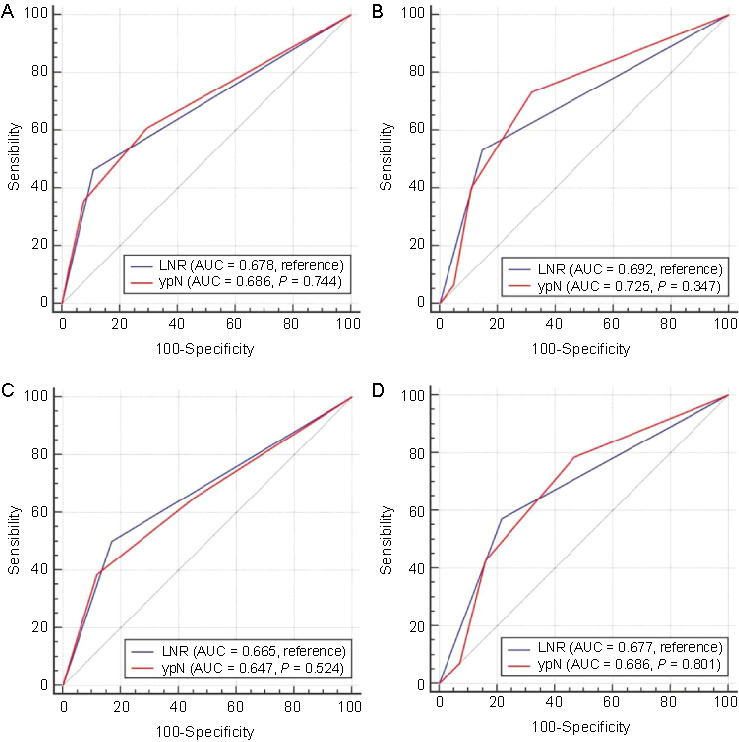
**The comparisons of the predictive values between LNR and ypN stage**. (A) The comparison of the predictive values between LNR and ypN stage for DFS in all cohorts; (B) The comparison of the predictive values between LNR and ypN stage for OS in all cohorts; (C) The comparison of the predictive values between LNR and ypN stage for DFS in non-PCR cohorts; (D) The comparison of the predictive values between LNR and ypN stage for OS in non-PCR cohorts. LNR showcased a prognostic ability for DFS and OS that was similar to that of the ypN stage. LNR: Lymph node ratio; ypN: Post-neoadjuvant therapy pathologic lymph nodes staging; DFS: Disease-free survival; OS: Overall survival; PCR: Pathological complete response; AUC: Area under the curve.

**Figure 5.**
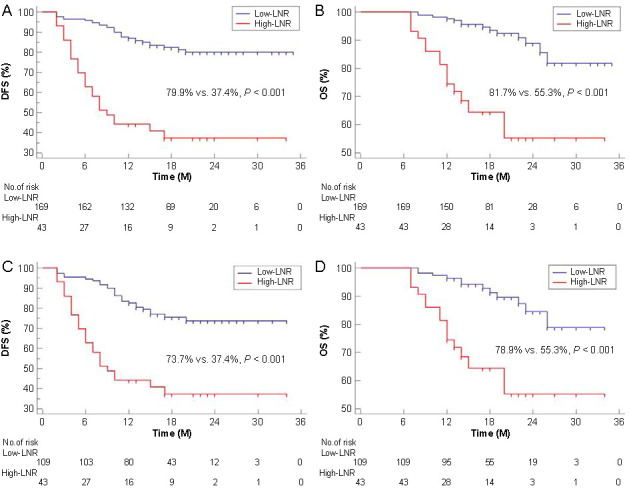
**The outcomes of patients grouped by their LNR values.** The Kaplan–Meier curves for DFS (A) and OS (B) in all cohorts, grouped by their LNR values. The Kaplan–Meier curves for DFS (C) and OS (D) in non-PCR cohorts, grouped by their LNR values. The patients were categorized into two groups based on their LNR values using the optimal cut-off value of 10%, as determined by the X-tile software. These groups were defined as the High-LNR group (with LNR ranging from 10% to 100%) and the Low-LNR group (with LNR ranging from 0% to 10%). A better 3-year OS (81.7% vs 55.3%; *P* < 0.001) and DFS (79.9% vs 37.4%; *P* < 0.001) was observed in patients with low LNR values. A similar result was also found in those with non-PCR, where the 3-year DFS was 73.7% vs 37.4% (*P* < 0.001), and the 3-year OS was 78.9% vs 55.3% (*P* < 0.001), between the groups. LNR: Lymph node ratio; DFS: Disease-free survival; OS: Overall survival; PCR: Pathological complete response.

The predictive value of LNR for DFS and OS is shown in Figure 3. According to the ROC curves, the AUCs for survival prediction were 0.688 (95% CI 0.621–0.750) for DFS and 0.733 (95% CI 0.668–0.791) for OS in ESCC after NICT. Similarly, subgroup analysis in non-PCR also indicated that LNR had good predictive values for OS (AUC ═ 0.699) and DFS (AUC ═ 0.651). To better understand its prognostic role, the prognostic values of LNR and ypN stage were compared. LNR showcased a prognostic ability for DFS and OS that was similar to that of the ypN stage (Figure 4). The results of the current study indicated that LNR could serve as an alternative to ypN stage for prognostication in ESCC after NICT. Figure 5 shows the outcomes categorized by the LNR values. A better 3-year OS (81.7% vs 55.3%; *P* < 0.001) and DFS (79.9% vs 37.4%; *P* < 0.001) was observed in patients with low LNR. A similar result was also found in those with non-PCR, where the 3-year DFS was 73.7% vs 37.4% (*P* < 0.001), and the 3-year OS was 78.9% vs 55.3% (*P* < 0.001), between the groups.

### Predictors of overall survival and disease-free survival

The results from the univariate and multivariate Cox analyses of prognostic factors associated with DFS and OS are shown in Tables 2 and 3. Both LNR and ypN stage were similarly determined by the number of positive LNs. To avoid collinearity issues, we employed two separate models (LNR and ypN stage) for the multivariate Cox analyses. The results indicated that LNR was an independent prognostic factor for both DFS (HR 4.013, 95% CI 2.259–7.127; *P* < 0.001) and OS (HR 7.026, 95% CI 3.203–15.413; *P* < 0.001). These results suggest that patients with a high LNR face a 4.013-fold increased risk of relapse (DFS) and a 7.026-fold elevated risk of death (OS). Additionally, the immunotherapy regimen did not emerge as an independent prognostic factor. The subgroup analysis revealed similar findings for non-PCR patients in terms of both DFS and OS (Table 4).

**Table 2:** Univariate analyses of prognostic factors in ESCC after the NICT

	**DFS**		**OS**	
	**HR (95% CI)**	***P* value**	**HR (95% CI)**	***P* value**
Sex (male vs female)	1.366 (0.494 – 3.778)	0.547	0.847 (0.257 – 2.796)	0.785
Age, years (>70 vs ≤70)	0.446 (0.191 – 1.040)	0.062	0.418 (0.127 – 1.379)	0.152
ECOG score (1 vs 0)	0.929 (0.421 – 2.052)	0.856	1.373 (0.525 – 3.590)	0.518
Smoking history (yes vs no)	0.930 (0.530 – 1.631)	0.800	0.929 (0.424 – 2.034)	0.854
Drinking history (yes vs no)	1.203 (0.666 – 2.174)	0.540	1.488 (0.638 – 3.474)	0.358
BMI, kg/m^2^ (>22.5 vs ≤22.5)	0.937 (0.519 – 1.694)	0.830	0.520 (0.199 – 1.360)	0.183
Tumor location		0.002		0.047
- Upper	Reference		Reference	
- Middle	0.279 (0.137 – 0.569)	<0.001	0.311 (0.119 – 0.814)	0.017
- Lower	0.455 (0.218 – 0.953)	0.037	0.395 (0.143 – 1.094)	0.074
Differentiation		0.813		0.14
- Well	Reference		Reference	
- Moderate	1.271 (0.573 – 2.819)	0.555	3.692 (0.855 – 15.931)	0.080
- Poor	1.285 (0.575 – 2.874)	0.541	2.258 (0.494 – 10.315)	0.293
Vessel invasion (yes vs no)	3.841 (2.088 – 7.068)	<0.001	2.195 (0.829 – 5.813)	0.114
Perineural invasion (yes vs no)	1.735 (0.933 – 3.228)	0.082	2.352 (1.075 – 5.145)	0.032
Tumor length, cm (>3 vs ≤3)	2.281 (1.309 – 3.973)	0.004	1.971 (0.897 – 4.331)	0.091
Surgical procedure (OE vs MIE)	0.759 (0.344 – 1.677)	0.496	0.342 (0.081 – 1.440)	0.144
ypT stage		0.003		0.029
- T0	Reference		Reference	
- T1-2	3.558 (1.436 – 8.819)	0.006	3.015 (0.829 – 10.967)	0.094
- T3-4a	4.595 (1.907 – 11.072)	0.001	5.030 (1.473 – 17.180)	0.010
ypN stage		<0.001		<0.001
- N0	Reference		Reference	
- N1	1.845 (0.929 – 3.663)	0.080	3.170 (1.188 – 8.462)	0.021
- N2-3	6.147 (3.363 – 11.234)	<0.001	9.735 (4.064 – 23.314)	<0.001
Immunotherapy regimen				0.838
- Camrelizumab	Reference		Reference	
- Nivolumab	0.541 (0.130 – 2.257)	0.399	0.468 (0.062 – 3.515)	0.461
- Pembrolizumab	1.008 (0.464 – 2.187)	0.985	0.889 (0.301 – 2.628)	0.831
- Sintilimab	0.627 (0.192 – 2.049)	0.440	1.097 (0.323 – 3.727)	0.882
- Tislelizumab	0.924 (0.466 – 1.835)	0.822	0.592 (0.199 – 1.759)	0.345
PCR (yes vs no)	0.167 (0.060 – 0.462)	0.001	0.175 (0.042 – 0.735)	0.017
LNR (high vs low)	5.131 (3.020 – 8.718)	<0.001	5.968 (2.901 – 12.277)	<0.001

DFS: Disease-free survival; OS: Overall survival; ESCC: Esophageal squamous cell carcinoma; NICT: Neoadjuvant immunochemotherapy; HR: Hazard ratio; ECOG: Eastern Cooperative Oncology Group; BMI: Body mass index; OE: Open esophagectomy; MIE: Minimally invasive esophagectomy; ypT: Post-neoadjuvant therapy pathologic primary tumor staging; ypN: Post-neoadjuvant therapy pathologic lymph nodes staging; PCR: Pathological complete response; LNR: Lymph node ratio.

**Table 3:** Multivariate analyses of prognostic factors in ESCC after the NICT

		**DFS**		**OS**	
		**HR (95% CI)**	***P* value**	**HR (95% CI)**	***P* value**
LNR model	Tumor location		0.002		0.04
	- Upper	Reference		Reference	
	- Middle	0.290 (0.141 – 0.595)	0.001	0.402 (0.151 – 1.066)	0.067
	- Lower	0.315 (0.146 – 0.679)	0.003	0.257 (0.089 – 0.741)	0.012
	PCR (yes vs no)	0.276 (0.096 – 0.792)	0.017		
	LNR (high vs low)	4.013 (2.259 – 7.127)	<0.001	7.026 (3.203 – 15.413)	<0.001
ypN model	Tumor location		0.004		0.024
	- Upper	Reference		Reference	
	- Middle	0.309 (0.151 – 0.633)	0.001	0.393 (0.149 – 1.039)	0.06
	- Lower	0.330 (0.154 – 0.710)	0.005	0.228 (0.078 – 0.660)	0.006
	PCR (yes vs no)	0.264 (0.089 – 0.781)	0.016		
	ypN stage		<0.001		<0.001
	N0	Reference		Reference	
	N1	1.189 (0.578 – 2.447)	0.639	3.236 (1.231 – 8.987)	0.018
	N2-3	4.199 (2.144 – 8.226)	<0.001	12.463 (4.895 – 31.732)	<0.001

The results indicated that LNR was an independent prognostic factor for both DFS (HR 4.013, 95% CI 2.259–7.127; *P* < 0.001) and OS (HR 7.026, 95% CI 3.203–15.413; *P* < 0.001). These results suggest that patients with a high LNR face a 4.013-fold increased risk of relapse (DFS) and a 7.026-fold elevated risk of death (OS). DFS: Disease-free survival; OS: Overall survival; ESCC: Esophageal squamous cell carcinoma; NICT: Neoadjuvant immunochemotherapy; HR: Hazard ratio; PCR: Pathological complete response; LNR: Lymph node ratio; ypN: Post-neoadjuvant therapy pathologic lymph nodes staging.

**Table 4:** Multivariate analyses for DFS and OS in non-PCR ESCC cohort, after the NICT

		**HR (95% CI)**	***P* value**
DFS	Tumor location		0.005
	- Upper	Reference	
	- Middle	0.303 (0.143 – 0.643)	0.002
	- Lower	0.326 (0.146 – 0.726)	0.006
	LNR (high vs low)	4.012 (2.255 – 7.140)	<0.001
OS	LNR (high vs low)	4.459 (2.101 – 9.462)	<0.001

DFS: Disease-free survival; OS: Overall survival; ESCC: Esophageal squamous cell carcinoma; NICT: Neoadjuvant immunochemotherapy; HR: Hazard ratio; PCR: Pathological complete response; LNR: Lymph node ratio.

## Discussion

The aim of this study was to evaluate the clinical impact of LNR in ESCC patients who underwent radical resection after NICT. The major result revealed that LNR serves as a significant independent predictor in prognostication. Furthermore, when prognosis was stratified by therapeutic response (PCR or non-PCR), LNR has also served as an independent predictor, showcasing a better discrimination ability in those with non-PCR. LNR had the similar prognostic ability for DFS and OS. Therefore, LNR might be a useful tool for assessing the LN status and could be considered as an alternative to the current ypN stage in ESCC patients receiving NICT.

The prognostic value of LNR has been analyzed in a variety of cancers, including EC [16–18]. A study including 353 Chinese EC patients who underwent radical resection reported that LNR, which demonstrated a superior predictive power than the pathologic nodal stage (pN) based on ROC analysis, was an independent risk factor for OS [17]. Another study also confirmed the prognostic value of LNR concerning survival in 120 EC patients who had curative surgery [18]. Moreover, the prognostic value of LNR has also been confirmed in EC after NAT [19, 20]. One study involving 199 ESCC patients who underwent radical resection post-NCT indicated that LNR’s prognostic accuracy was generally superior to the pN stage [19]. Another study, which included 7195 EC patients from the National Cancer Database who received NCRT, found that LNR outperformed ypN in predicting OS for EC patients after NCRT [20]. In the current study, LNR, as well as ypN stage, were confirmed as independent predictors for both OS and DFS. The ROC curve revealed a good predictive value of LNR in both DFS and OS, and the predictive value was similar to the ypN stage. Therefore, LNR could be regarded as an alternative to the ypN stage when prognosticating outcomes for ESCC patients who have undergone NICT.

Although the prognostic value of LNR has been confirmed, the optimal threshold for LNR remains inconsistent. Most of the published studies have determined the optimal threshold of LNR using the ROC curve or X-tile software based on prognosis [17–20]. One study, which included 536 EC patients who underwent curative resection, was conducted to investigate the prognostic significance of LNR, had a set cut-off value of 0.2 determined by the ROC analysis [28]. Moreover, another study, which included 120 EC patients who underwent radical resection, suggested that the optimal LNR cut-off value was 10% based on the ROC analysis [18]. In addition, another study, which involved 199 patients with locally advanced ESCC who underwent radical resection after NCT, revealed that 13% was the optimal cut-off value, as identified by the X-tile software [19]. Several studies have shown that the PCR after NAT is a good predictor in evaluating the long-term survival in a variety of cancers [29, 30]. A growing body of evidence also confirms that patients achieving PCR could truly benefit from NAT. Otherwise, for patients with non-PCR, the prognosis might be poorer than for those who undergo surgery alone, given the postoperative complications and the toxicity associated with NAT [31]. Therefore, in the current study, the optimal cut-off value of LNR was determined using the X-tile software, with PCR as the dependent variable.

It has previously been reported that fewer TLNs are removed during dissection in ESCC patients who undergo NAT compared to those who do not receive NAT [32]. Furthermore, the LNR is believed to address the potential bias related to an insufficient number of TLNs. There are some differences between the present study and previous studies. Firstly, the treatment methods varied. Most previous studies analyzed the patients who either only underwent surgery alone or had surgery with perioperative NCT or NCRT. In the present study, ESCC patients who underwent surgery after NICT were analyzed. Secondly, the median numbers of TLNs were different, which might affect the cut-off value of LNR.

The present study has some limitations. Firstly, it was a retrospective research conducted at a single institution. Secondly, there may be a patient selection bias in the study. Thirdly, a temporal bias might be present, as surgical procedures, perioperative care, and adjuvant treatments changed over the course of the study. Fourthly, the follow-up duration in the current study was too short, which could introduce bias when predicting prognostic parameters. Therefore, these results should be confirmed in future prospective multicenter studies.

## Conclusion

To the best of our knowledge, this is the first study to investigate the prognostic value of LNR in ESCC patients who underwent radical resection following NICT. While the duration of follow-up in this study is short, LNR has emerged as a significant predictor for survival in ESCC patients who underwent radical surgery post-NICT. This real-world data could provide valuable evidence to guide effective postoperative adjuvant therapy for ESCC patients after the NICT. Consequently, LNR may serve as a useful tool for assessing the LN status and might be considered as an alternative to the ypN stage for ESCC patients undergoing NICT.

## References

[1] Sung H, Ferlay J, Siegel RL, Laversanne M, Soerjomataram I, Jemal A (2021). Global cancer statistics 2020: GLOBOCAN estimates of incidence and mortality worldwide for 36 cancers in 185 countries. CA Cancer J Clin.

[2] Cao W, Chen HD, Yu YW, Li N, Chen WQ (2021). Changing profiles of cancer burden worldwide and in China: a secondary analysis of the global cancer statistics 2020. Chin Med J (Engl).

[3] van Hagen P, Hulshof MC, van Lanschot JJ, Steyerberg EW, van Berge Henegouwen MI, Wijnhoven BP (2012). Preoperative chemoradiotherapy for esophageal or junctional cancer. N Engl J Med.

[4] Ando N, Kato H, Igaki H, Shinoda M, Ozawa S, Shimizu H (2012). A randomized trial comparing postoperative adjuvant chemotherapy with cisplatin and 5-fluorouracil versus preoperative chemotherapy for localized advanced squamous cell carcinoma of the thoracic esophagus (JCOG9907). Ann Surg Oncol.

[5] Hou S, Pan Z, Hao X, Hang Q, Ding Y (2021). Recent progress in the neoadjuvant treatment strategy for locally advanced esophageal cancer. Cancers (Basel).

[6] Kojima T, Shah MA, Muro K, Francois E, Adenis A, Hsu CH (2020). Randomized phase III KEYNOTE-181 study of pembrolizumab versus chemotherapy in advanced esophageal cancer. J Clin Oncol.

[7] Kato K, Cho BC, Takahashi M, Okada M, Lin CY, Chin K (2019). Nivolumab versus chemotherapy in patients with advanced oesophageal squamous cell carcinoma refractory or intolerant to previous chemotherapy (ATTRACTION-3): a multicentre, randomised, open-label, phase 3 trial. Lancet Oncol.

[8] Shen D, Chen Q, Wu J, Li J, Tao K, Jiang Y (2021). The safety and efficacy of neoadjuvant PD-1 inhibitor with chemotherapy for locally advanced esophageal squamous cell carcinoma. J Gastrointest Oncol.

[9] Wu Z, Zheng Q, Chen H, Xiang J, Hu H, Li H (2021). Efficacy and safety of neoadjuvant chemotherapy and immunotherapy in locally resectable advanced esophageal squamous cell carcinoma. J Thorac Dis.

[10] Hong ZN, Weng K, Peng K, Chen Z, Lin J, Kang M (2021). Neoadjuvant immunotherapy combined chemotherapy followed by surgery versus surgery alone for locally advanced esophageal squamous cell carcinoma: a propensity score-matched study. Front Oncol.

[11] Yang G, Su X, Yang H, Luo G, Gao C, Zheng Y (2021). Neoadjuvant programmed death-1 blockade plus chemotherapy in locally advanced esophageal squamous cell carcinoma. Ann Transl Med.

[12] Rice TW, Ishwaran H, Hofstetter WL, Kelsen DP, Apperson-Hansen C, Blackstone EH (2016). Recommendations for pathologic staging (pTNM) of cancer of the esophagus and esophagogastric junction for the 8th edition AJCC/UICC staging manuals. Dis Esophagus.

[13] Chen YJ, Yeh ST, Kao PS, Ou LH, Lin CS (2020). A reappraisal of lymph node dissection in colorectal cancer during primary surgical resection. World J Surg Oncol.

[14] Tochigi K, Nagayama J, Bando S, Ishiyama A, Yuba T, Yuguchi Y (2022). Relationship between the number of lymph nodes dissected and prognosis in muscle-invasive bladder cancer in the era of neoadjuvant chemotherapy. Int J Urol.

[15] Cipolla C, Galvano A, Vieni S, Saputo F, Lupo S, Latteri M (2021). Effects of the number of removed lymph nodes on survival outcome in patients with sentinel node-negative breast cancer. World J Surg Oncol.

[16] Tan Z, Ma G, Yang H, Zhang L, Rong T, Lin P (2014). Can lymph node ratio replace pN categories in the tumor-node-metastasis classification system for esophageal cancer?. J Thorac Oncol.

[17] He Z, Wu S, Li Q, Lin Q, Xu J (2013). Use of the metastatic lymph node ratio to evaluate the prognosis of esophageal cancer patients with node metastasis following radical esophagectomy. PLoS One.

[18] Yukawa N, Aoyama T, Tamagawa H, Tamagawa A, Atsumi Y, Kawahara S (2020). The lymph node ratio is an independent prognostic factor in esophageal cancer patients who receive curative surgery. In Vivo.

[19] Kano K, Yamada T, Komori K, Watanabe H, Takahashi K, Fujikawa H (2021). The prognostic value of lymph node ratio in locally advanced esophageal cancer patients who received neoadjuvant chemotherapy. Ann Surg Oncol.

[20] Zhang Y, Cao Y, Zhang J, Huang M, Roy P, Huang B, et al.

[21] Zhang T, Hou X, Li Y, Fu X, Liu L, Xu L (2020). Effectiveness and safety of minimally invasive Ivor Lewis and McKeown oesophagectomy in Chinese patients with stage IA-IIIB oesophageal squamous cell cancer: a multicentre, non-interventional and observational study. Interact Cardiovasc Thorac Surg.

[22] Brown AM, Pucci MJ, Berger AC, Tatarian T, Evans NR 3rd, Rosato EL (2018). A standardized comparison of peri-operative complications after minimally invasive esophagectomy: Ivor Lewis versus McKeown. Surg Endosc.

[23] Dindo D, Demartines N, Clavien PA (2004). Classification of surgical complications: a new proposal with evaluation in a cohort of 6336 patients and results of a survey. Ann Surg.

[24] Chirieac LR, Swisher SG, Ajani JA, Komaki RR, Correa AM, Morris JS (2005). Posttherapy pathologic stage predicts survival in patients with esophageal carcinoma receiving preoperative chemoradiation. Cancer.

[25] Kelly RJ, Ajani JA, Kuzdzal J, Zander T, Van Cutsem E, Piessen G (2021). Adjuvant nivolumab in resected esophageal or gastroesophageal junction cancer. N Engl J Med.

[26] Li J, Qiu R, Hu Y, Wang Y, Qi Z, He M, Li Y (2021). Postoperative adjuvant therapy for patients with pN+ esophageal squamous cell carcinoma. Biomed Res Int.

[27] Li L, Zhao L, Lin B, Su H, Su M, Xie D (2017). Adjuvant therapeutic modalities following three-field lymph node dissection for stage II/III esophageal squamous cell carcinoma. J Cancer.

[28] Mariette C, Piessen G, Briez N, Triboulet JP (2008). The number of metastatic lymph nodes and the ratio between metastatic and examined lymph nodes are independent prognostic factors in esophageal cancer regardless of neoadjuvant chemoradiation or lymphadenectomy extent. Ann Surg.

[29] Rosner S, Liu C, Forde PM, Hu C (2022). Association of pathologic complete response and long-term survival outcomes among patients treated with neoadjuvant chemotherapy or chemoradiotherapy for NSCLC: a meta-analysis. JTO Clin Res Rep.

[30] Schroeder W, Ghadimi MPH, Schloesser H, Loeser H, Schiller P, Zander T (2022). Long-term outcome after histopathological complete response with and without nodal metastases following multimodal treatment of esophageal cancer. Ann Surg Oncol.

[31] Murakami Y, Hamai Y, Emi M, Hihara J, Imano N, Takeuchi Y (2018). Long-term results of neoadjuvant chemoradiotherapy using cisplatin and 5-fluorouracil followed by esophagectomy for resectable, locally advanced esophageal squamous cell carcinoma. J Radiat Res.

[32] Hagens E, Tukanova K, Jamel S, van Berge Henegouwen M, Hanna GB, Gisbertz S (2022). Prognostic relevance of lymph node regression on survival in esophageal cancer: a systematic review and meta-analysis. Dis Esophagus.

